# Supplementary material for: Comprehensive omic characterization of breast cancer in Mexican-Hispanic women
Source: Nat Commun. 2021 Apr 14;12:2245. doi: 10.1038/s41467-021-22478-5 (PMC8046804; doi:10.1038/s41467-021-22478-5)
Supplement: Supplementary file 1 — Supplementary information [file 41467_2021_22478_MOESM1_ESM.pdf]

Supplementary figures

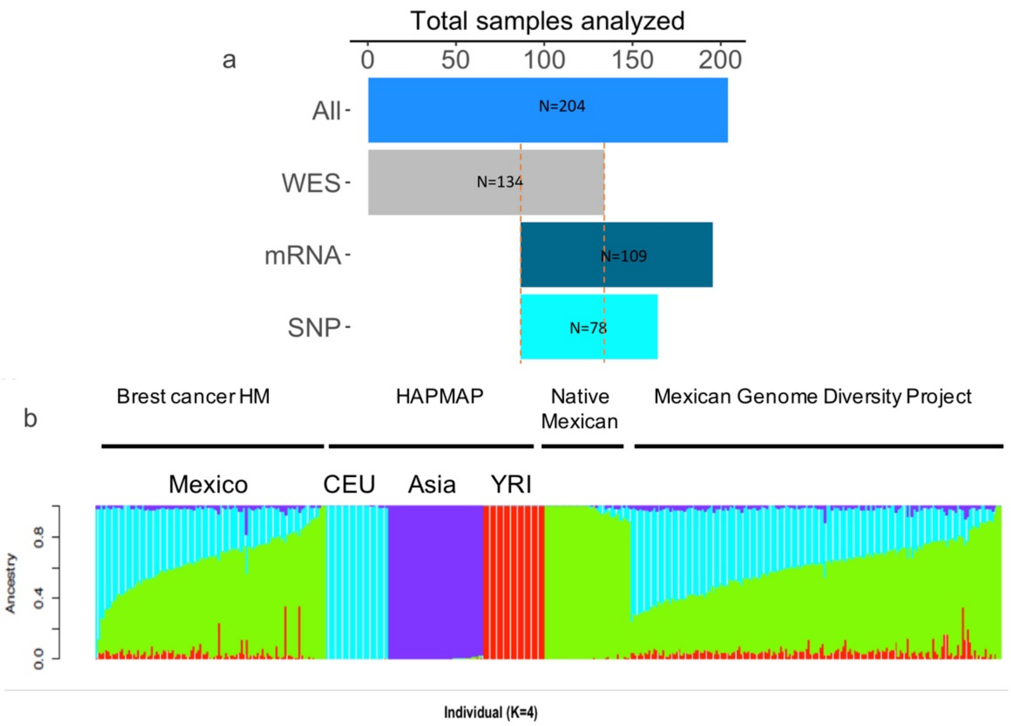

c

	Population	Samples	Details
**HapMap**		**95**	
	**CEU (27), CHB+JPT (41) and YRI (27)**		
**MGDP**		**161**	**From 7 states of Mexico**
	**GUA**	**26**	**Guanajuato**
	**GUE**	**27**	**Guerrero**
	**SON**	**24**	**Sonora**
	**TAM**	**9**	**Tamaulipas**
	**VER**	**26**	**Veracruz**
	**YUC**	**24**	**Yucatan**
	**ZAC**	**25**	**Zacatecas**
**Native Mexican**		**37**	
	**ZAP**		**Oaxaca (Center)**
	**MAY**		**Campeche (Southeast)**
	**TEP**		**Durango (North)**

Supplementary Fig. 1: HM tumors analyzed in our sample collection and estimation of genetic ancestry on HM data set. a) Total number of samples analyzed in each multiomic platform. Orange dot lines indicate share samples across different platforms b) Individual ancestry populations. Four parental groups K=4: Asia (CHB+JPT), Europe (CEU), Africa (YRI) and Native Mexican Zapoteca, Maya and Tepehuano (ZAP, MAY, TEPEH) and Breast cancer cases. c) Populations used for ancestry assessment. The ancestry proportions for the Mexican mestizos were as follow: 38 % European, 55 % Native American, 5% African and 1 % East Asia ancestry proportions. WES: Whole exome sequencing, SNP: Small nucleotide polymorphism, CEU: Utah Residents (CEPH) with Northern and Western European Ancestry, CHB: Han Chinese in Beijing, China, JPT: Japanese in Tokyo, Japan, YRI: Yoruba in Ibadan, Nigeria.

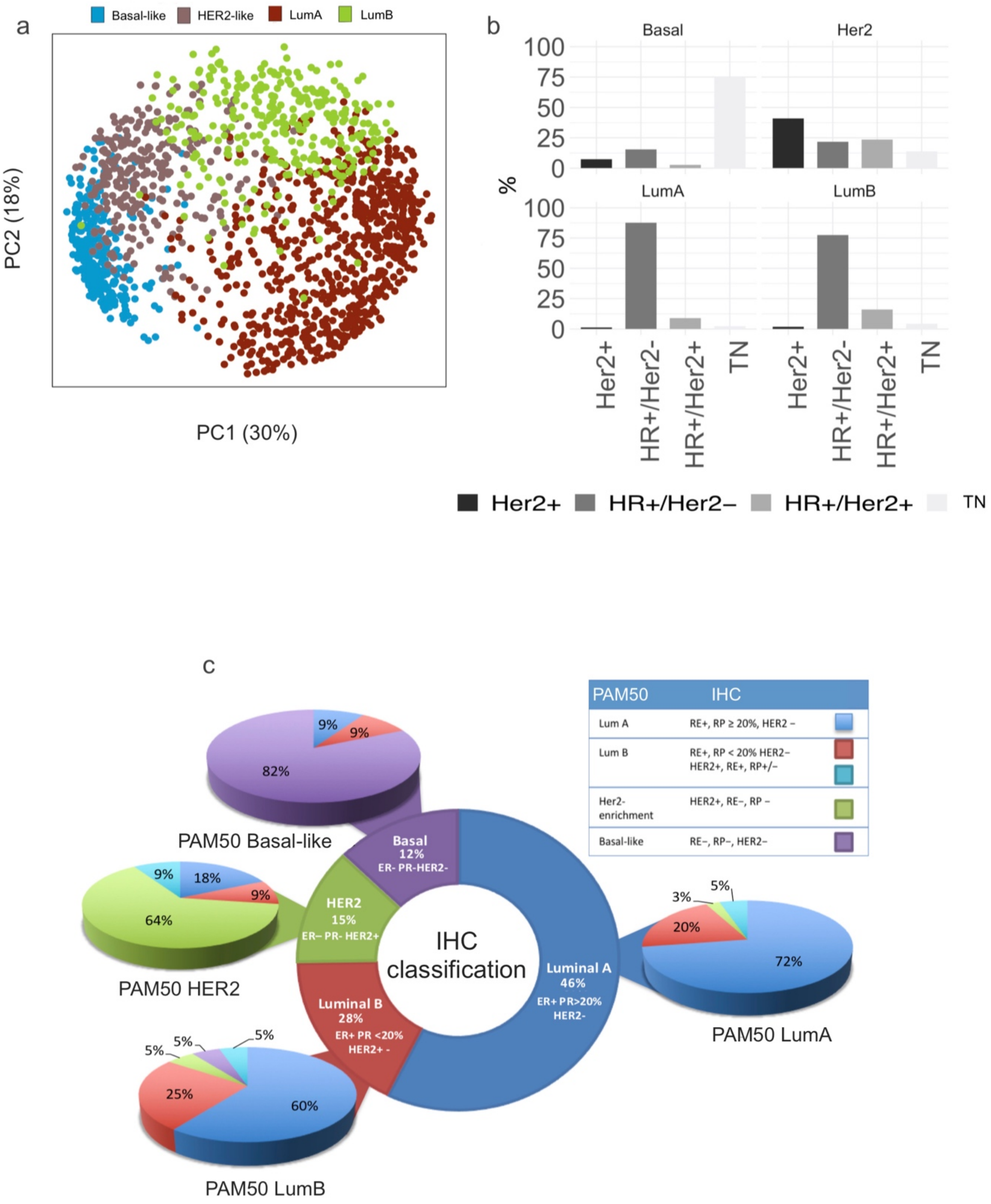

Supplementary Fig. 2: Immunochemical classification among PAM50 tumor subtypes. a) Principal component analysis shows the clustering of BC cases from 4 ancestry-groups into different tumor subtypes, as defined by the PAM50 classifier. b) Proportion of IHC markers presence among PAM50 tumor subtypes in all the evaluated ancestry-groups. c) Percentage of concordance between IHC tumor classification and their corresponding PAM50 subtype classification in HM data set**.** Main inner pie chart shows the partition of tumors into different IHC classes and for each partition, outer pie charts show their breakdown proportion into PAM50 subtypes. LumA: Luminal A, LumB: Luminal B. IHC: immunochemistry. ER: Estrogen receptor, PR: Progesterone receptor, TN. Triple negative (ER-, PR-, HER2-).

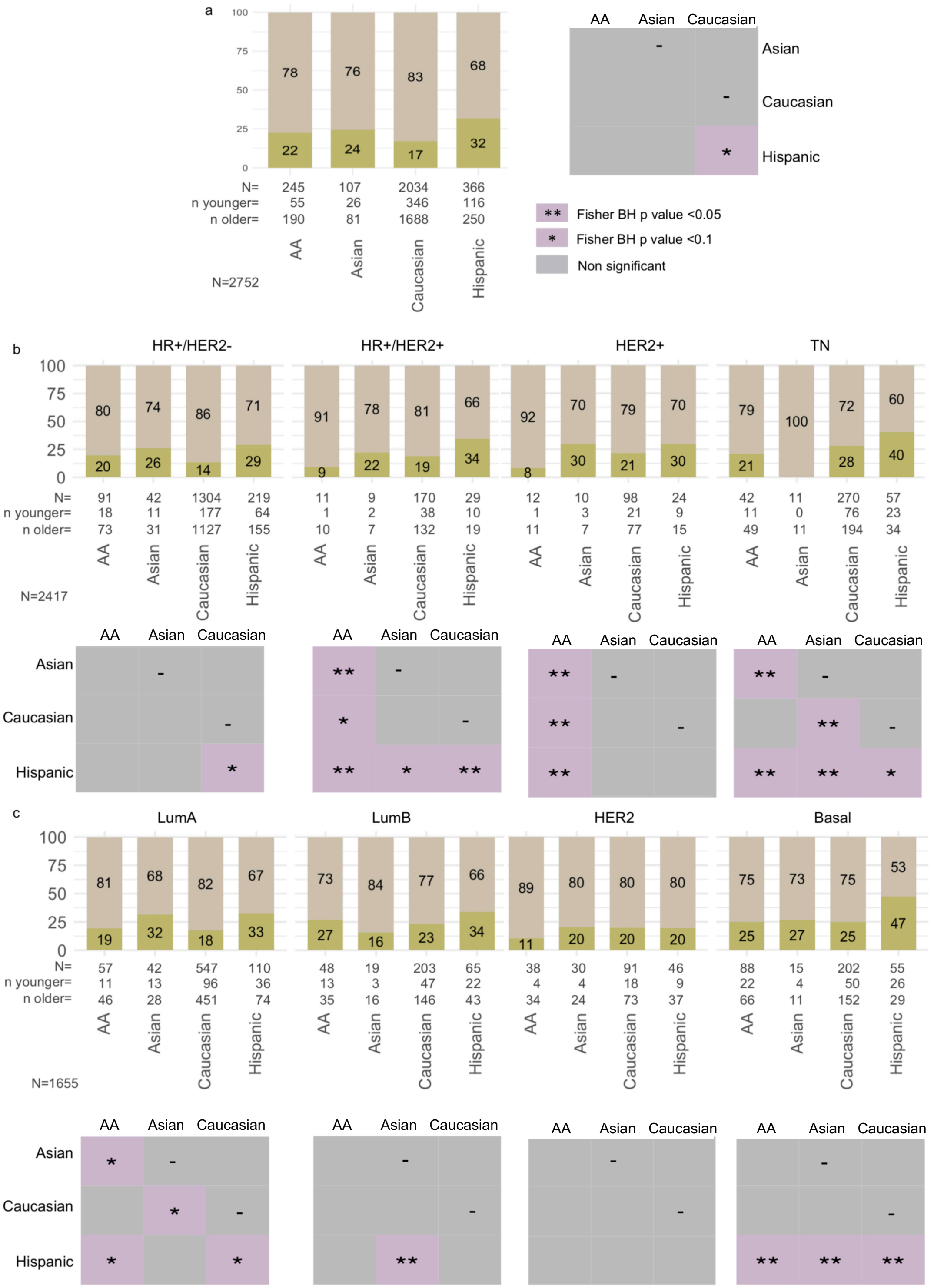

Supplementary Fig. 3: Age distribution in all BC samples and within IHC and molecular intrinsic subtypes within AA, Asian, Caucasian and Hispanic patients evaluated. a) Frequency of younger (≤45 years of age) and elderly (>45 years of age) BC patients among ancestries. Frequency of BC b) immunochemistry subtypes and c) PAM50 intrinsic molecular subtypes within patients from different ancestry diagnosis at early-age (≤45 years of age) or elderly-age (>45 years of age). Barplots represent proportion of age classes in each population group, while heatmaps represent the BH adjusted p-values computed by a two-sided Fisher's exact test from multiple comparisons. Corresponding p-values are reported on Supplementary Data 2. HM: Hispanic-Mexican, Hispanics: average value between HM and Hispanics non-Mexican evaluated tumors. BH adjusted p-value **<0.05, * <0.1. AA: African American. HR: Hormonal receptors (ER and PR).

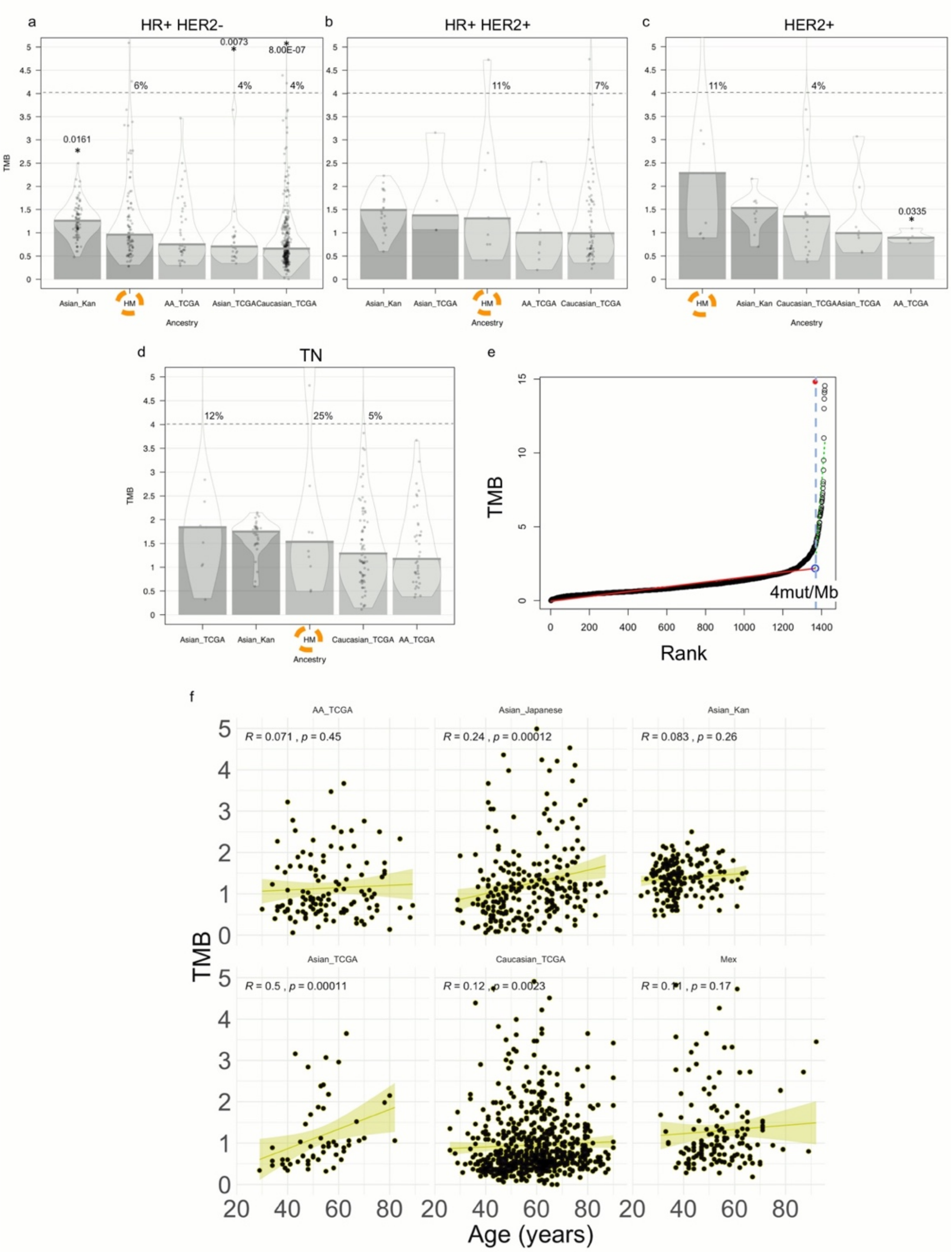

Supplementary Fig. 4: TMB scenario in breast cancer samples from different human populations. Violin plots showing the TMB (mut/Mb) distribution in each ancestry-group considering point non-silent mutation divided by IHC subtype: a) HR+/HER2-, b) HR+/HER2+, c)HER2, d) TN. Dot line represents hypermutated threshold (4mut/Mb) and the number indicate the percentage of hypermutated tumors in each data set. Statistical comparisons were assessed with a two-tailed Wilcoxon test considering HM dataset as reference. HM samples indicated by orange circle. e) Determination of a hypermutation cutoff in BC cancer through segmented linear regression analysis. Dotted lines indicate the point at which there was observed to be a significantly slope. f) Scatter plots showing the Pearson correlation (R) between TMB and age at diagnosis in breast cancer patients from different data sets and ancestries. On panel a-d, inserted barplots represent the median value, gray dots represent individual patient data.p-values are indicated near the corresponding asterisks. BH adjusted p-value *<0.05. On panel f p-values of correlation coefficients were computed using a two-tailed student t-test. AA: African American, HM: Hispanic Mexican, HR: Hormonal receptor (Estrogen an progesterone receptor).

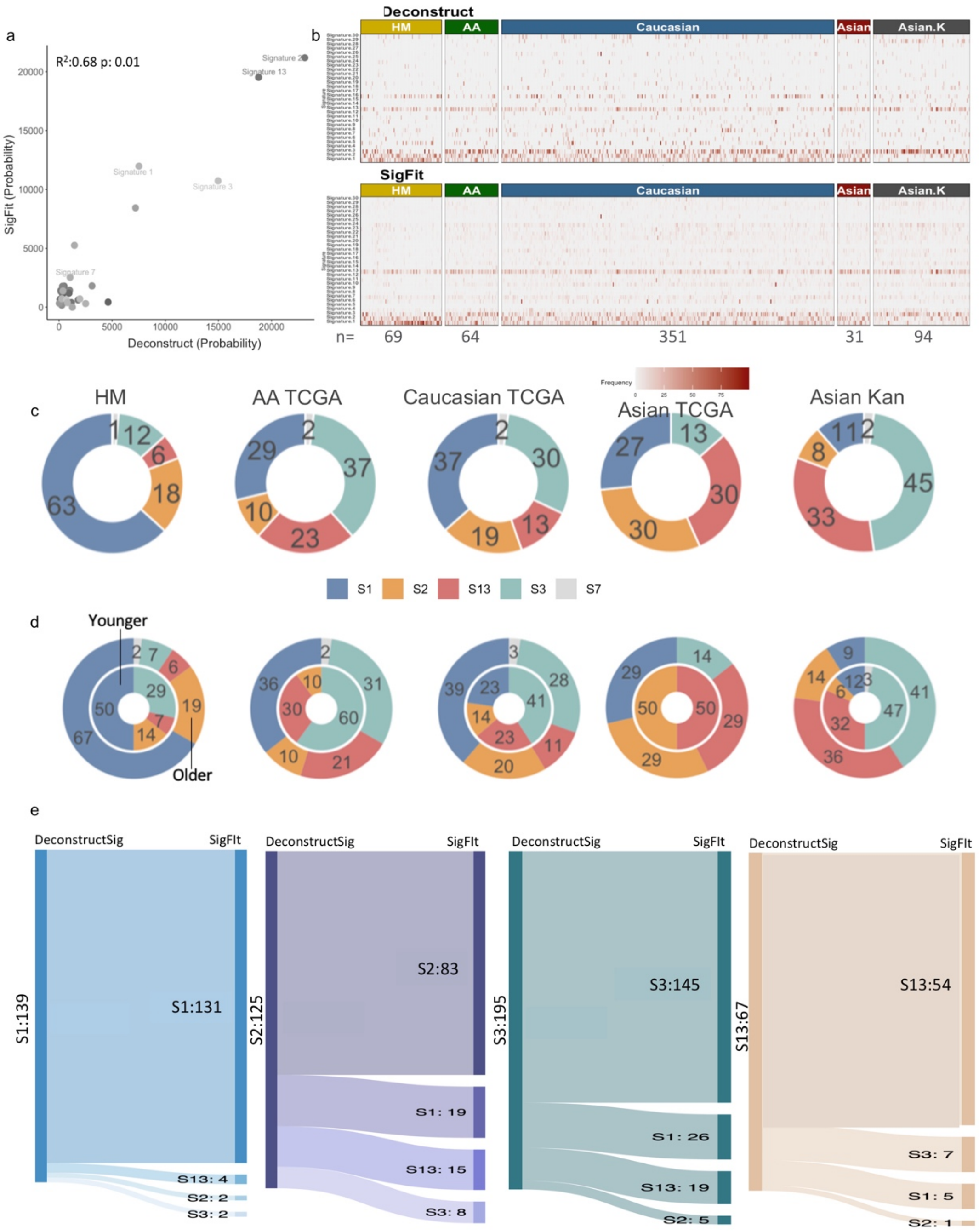

Supplementary Fig. 5: Mutational signatures profiles in BC samples from different populations. a) Correlation between probabilities (contributions) computed with two different approaches for signature decomposition (deconstructSig and Sigfit). R Pearson coefficients and two-tailed t-test p-values of correlation levels were estimated. Higher agreement between the two methods guarantees the optimal deconstruction of particular mutational process. b) Heatmap showing the individual contribution of the 30 COSMIC v2 mutational signatures in each tumor sample evaluated from diverse ancestry-groups and tumor collections. Color scale represents the level of contribution (Probability), from white, for the lowest contribution value, to red, for the highest contribution value. c) Normalized cumulative probabilities of top5 mutational signatures in the evaluated breast tumors belonging to diverse human populations deconstructed by SigFit. d) Donut plot showing the percentage of each top5 mutational signature in younger (≤45 years of age – Inner donut chart) and elderly patients (>45 years of age outer donut chart). e) Sankey plot showing the concordance between the deconstructed signatures by DeconstructSigs (left) and SigFit (right). The number represents the total sample in each mutational signature. S: signature.

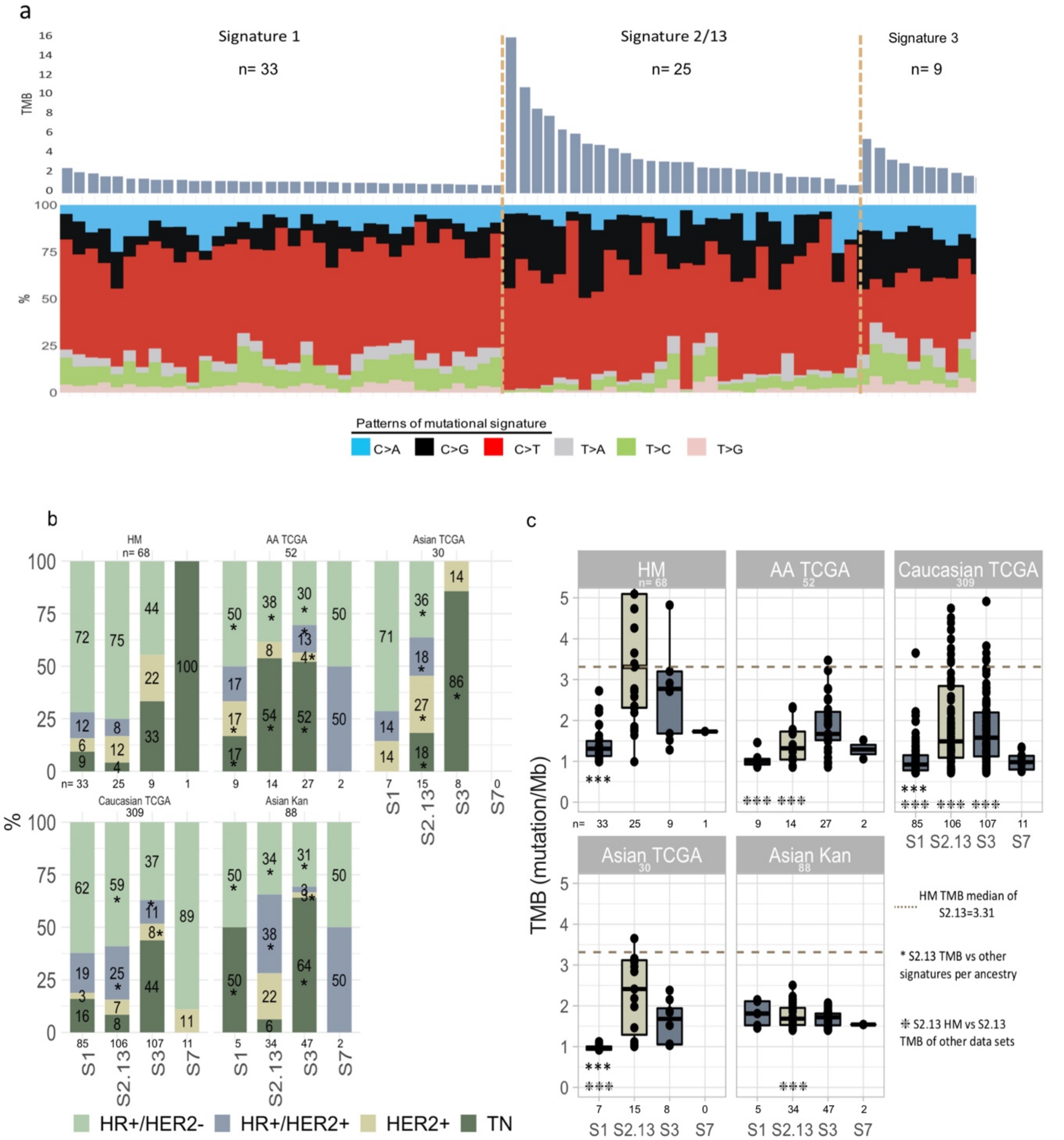

Supplementary Fig. 6: Mutational signatures of breast cancer a) Common plot showing the nucleotide substitution panorama in HM tumors across the most prevalent signatures (i.e., 1, 2/13 and 3). From top to bottom, barplot in the first panel summarizes TMB in tumors classified in each prevalent signature (i.e., with the highest contribution to individual tumors). The second panel shows the six types of nucleotide substitutions represented by different colors as illustrated in figure legend. b) Percentage of IHC tumor subtypes across the most prevalent signatures in the evaluated ancestry-groups. c) Boxplot showing the distribution of TMB (tumor mutational burden) in tumors from different ancestry and splited according to their most prevalent mutational signatures. Box plots in panel a-d represent median ± IQR (25th and 75th percentile) and whiskers correspond to maximum and minimum values. Significant differences (FDR<0.05) for indicated comparisons in panel b were computed by a two-sided Fisher exact test adjusting for multiple hypothesis by BH, while for panel c were calculated by a two-sided Wilcoxon rank-sum test and values adjusted by BH procedure. Corresponding p-values for panels b-c are reported on Supplementary Data 4. S: signature, HM: Hispanic Mexican. AA: African American. HR: hormonal receptors (estrogen and progesterone receptors).

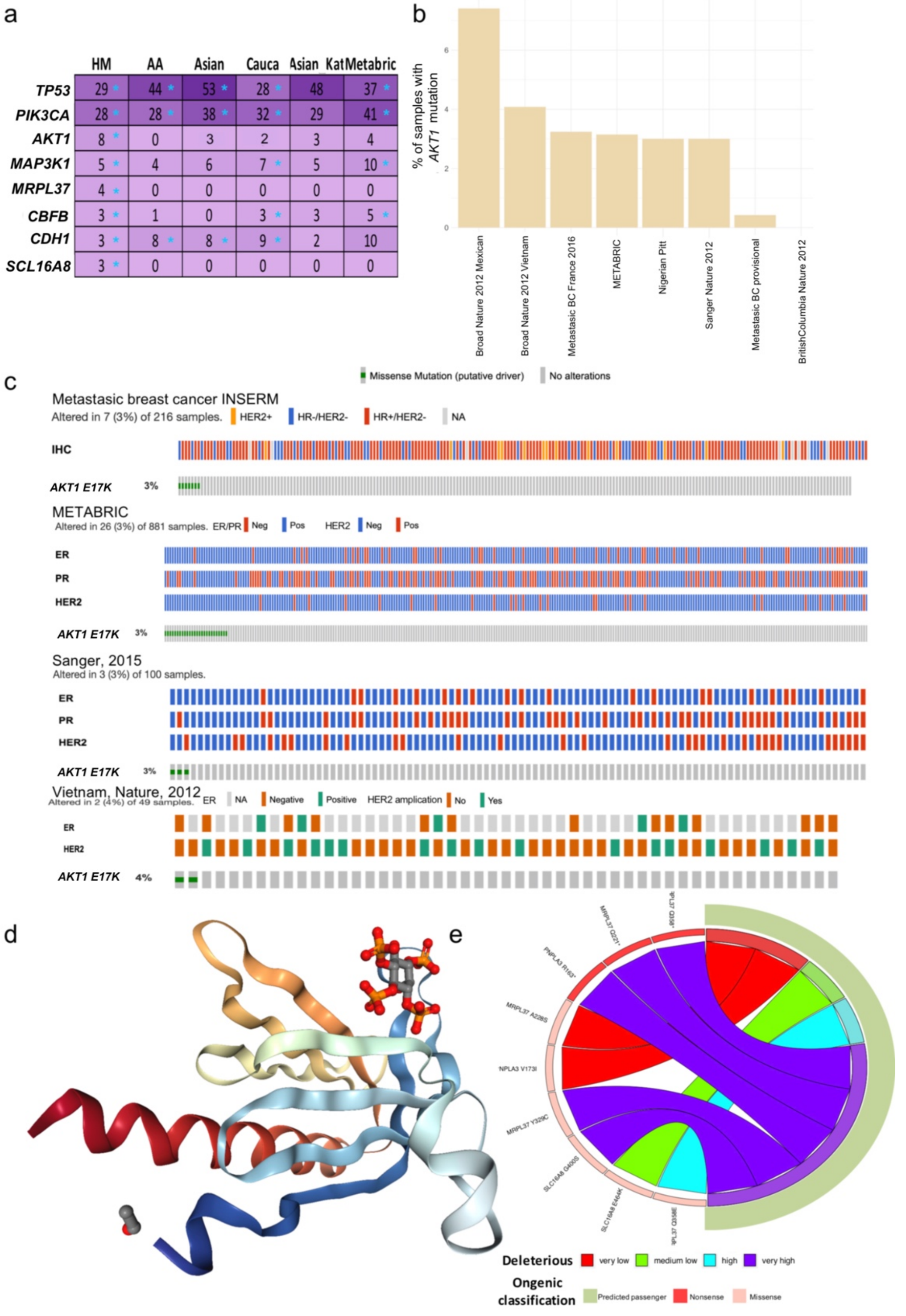

Supplementary Fig. 7: Somatic mutations significantly present in BC samples. a) Heatmap showing the percentage of samples with somatic mutations on top mutated genes from MutSigCV analysis in HM profiled tumors and different analyzed ancestry-groups. The percentage of samples presenting a mutation in each gene is shown inside the corresponding cell and highlighted in different shades of purple. * qvalue <0.1 by MutSig algorithm. b) Percentage of samples with mutations in AKT gene in different BC cohorts included in cbioportal. c) Oncoplot showing the percentage of AKT1 E17K mutation and immunochemistry makers status in publicly available BC data sets d) 3d structural view of AKT1 protein harboring E17K mutation. e) Circus plot of the oncogenic classification and deleterious capacity of novel mutations identified in HM cohort.

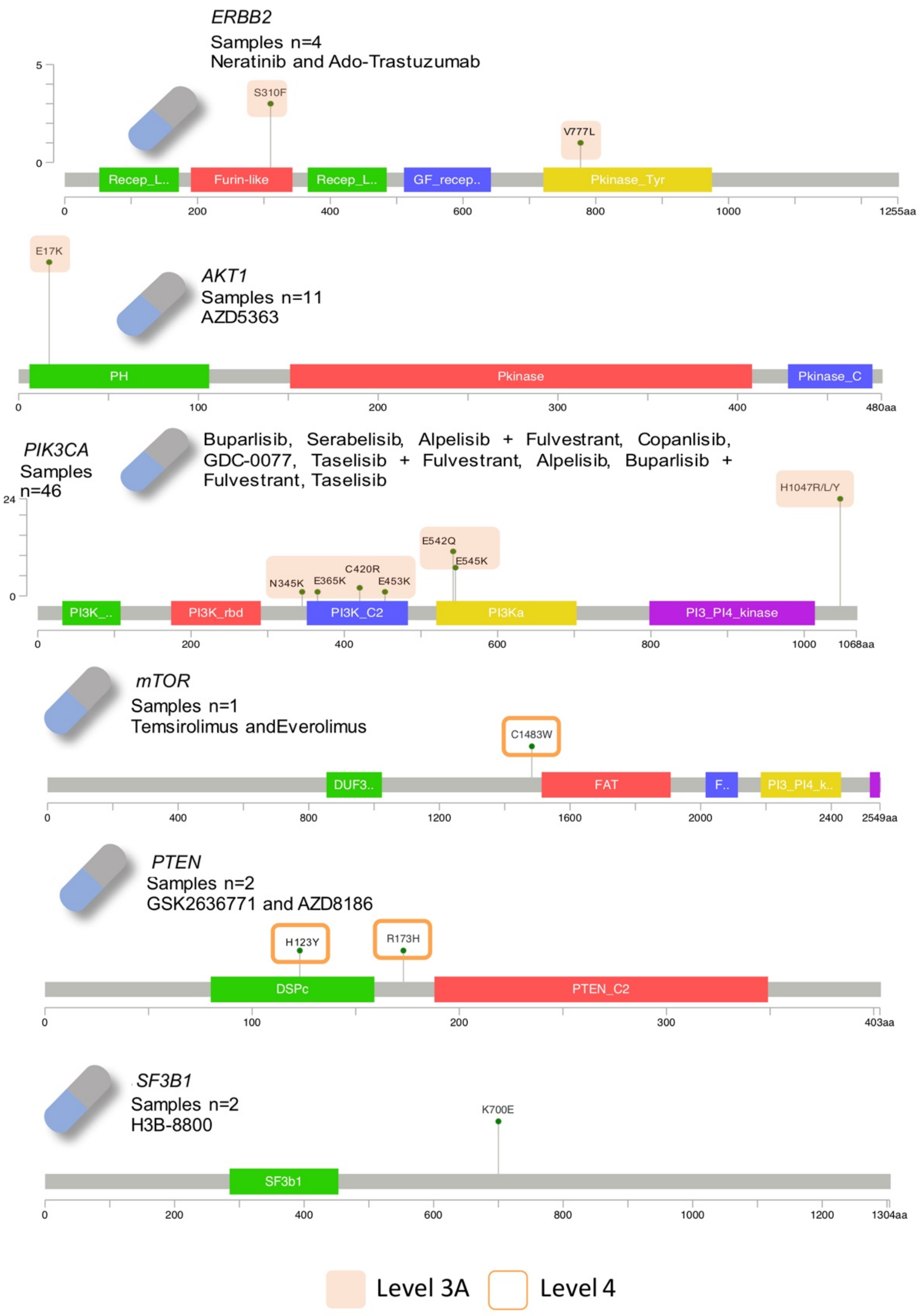

Supplementary Fig. 8: Somatic Mutations in actionable genes potentially druggable in HM cohort. Lollipop plots are the schematic representation of genes and their functional domains which show in detail the localization of point somatic mutations detected in actionable genes as identified by OncoKb including ERBB2, AKT1, PIK3CA, MTOR, PTEN and SF3B1divided according to the level of supporting evidence as follows: 3A (clinical Evidence, solid rectangle in orange) and 4 (Biological evidence, rectangle with orange outline). The height of the lollipop is proportional to the relative frequency of each mutation. Drugs with potentially activity against the genetic change are indicated next to the pill symbol.

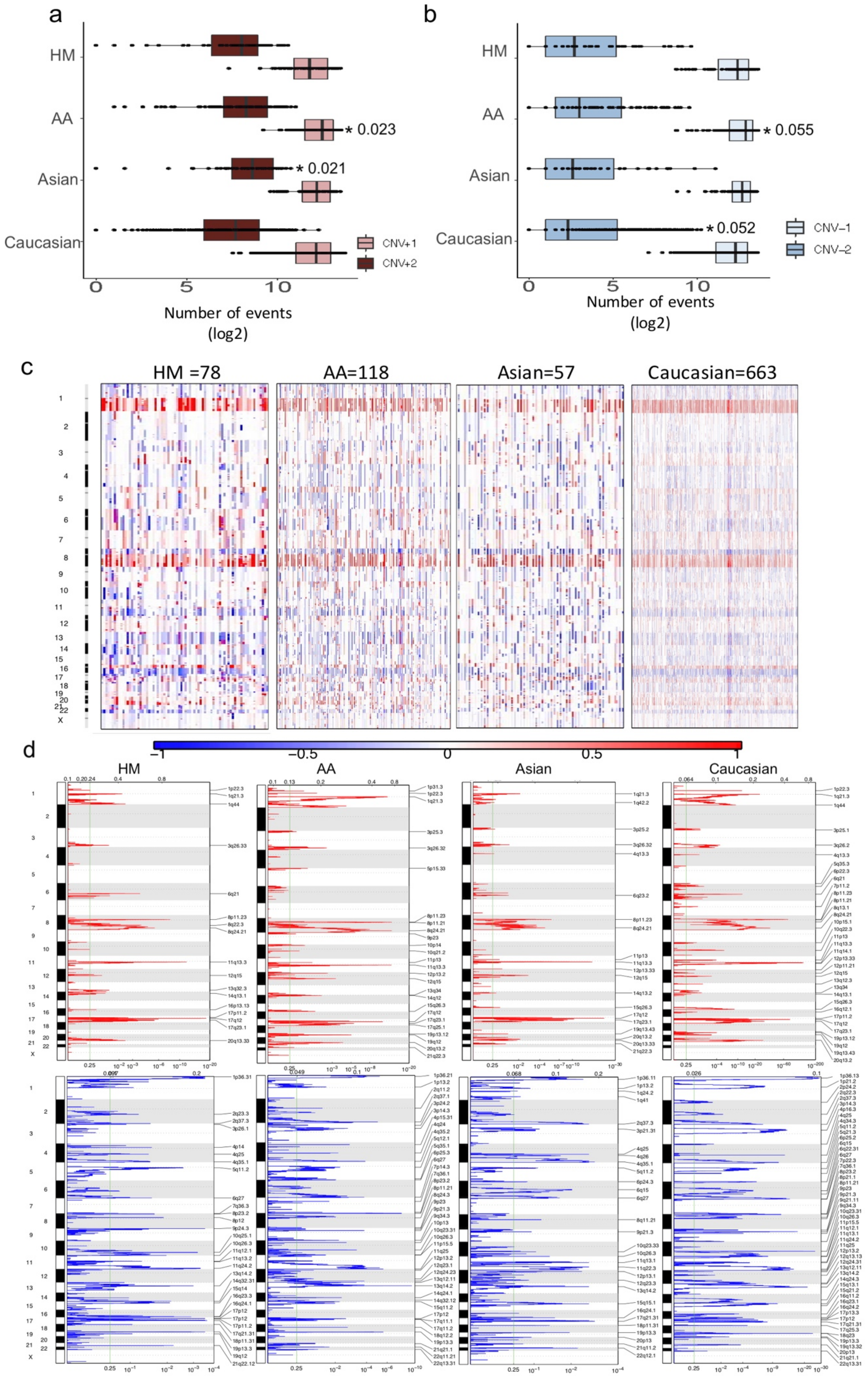

a
HM
AA
Asian
Caucasian
* 0.023
* 0.021
CNV+1
CNV+2
Number of events
(log2)
b
* 0.055
* 0.052
CNV−1
CNV−2
c
HM =78
AA=118
Asian=57
Caucasian=663
d

Supplementary Fig. 9: GISTIC analysis of copy number aberrations in BC samples. a) Distribution of total significant genome amplification events in each population (+1 likely significant as low-level copy number aberrations-, +2 significant among overall tumor analyzed as high-level copy number aberrations). b) Distribution of total significant genome deletion events in each population (+1 likely significant as low-level copy number aberrations-, +2 significant among overall tumor analyzed as high-level copy number aberrations). c) Heatmap showing segmented copy-number log2 (ratio) values across BC cases in each population. Chromosomes are vertically oriented from top to bottom and samples are arranged from left to right. d) GISTIC plot showing recurrent amplifications (top, red) and deletions (bottom, blue) across chromosomic regions (from top to bottom) in each population. X-axis shows GISTIC G scores (top) and q-values (bottom), with a green line representing the significant threshold (q-value =0.25). Candidate genes of interest with their corresponding genomic location are labeled on the right. Asterisk in a and b indicate significant differences (FDR<0.05) estimated with a two-tailed Wilcoxon rank-sum test. Box plots showing in panel a-b represent median ± IQR (25th and 75th percentile) and whiskers correspond to maximum and minimum values. HM: Hispanic Mexican, AA: African American.

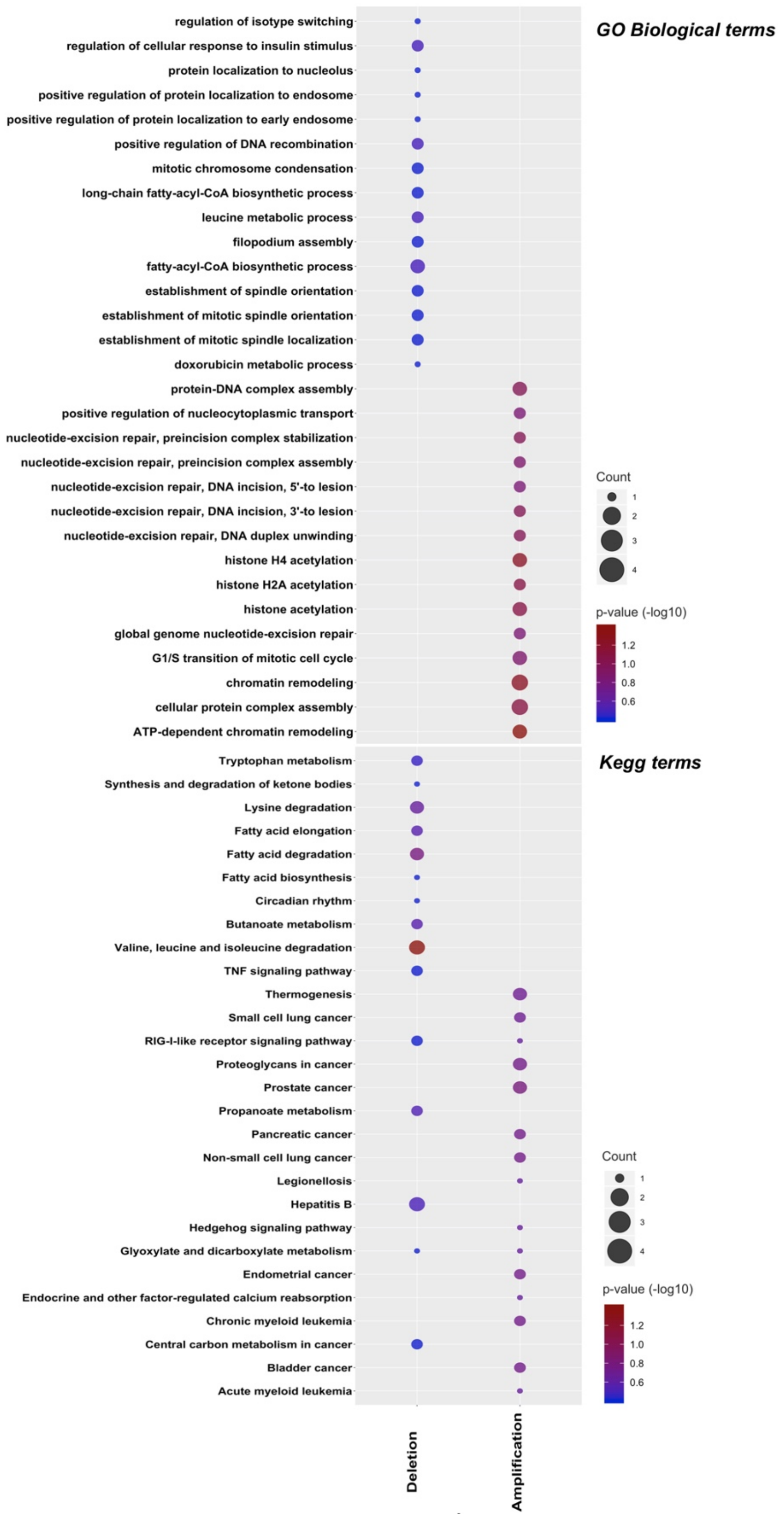

Supplementary Fig. 10: Gene set over-representation analysis for genes significantly correlated with SCNA in HM cohort. Bubble plot represents GO (top) or KEEG (bottom) terms significantly over-represented (p-value <0.05) in genes which expression level shows significant positive correlation with log2 segmented copy number values (Pearson > 0.3; p-value < 0.05) of focal amplifications or deletions in genomic regions in which they are encoded Bubble size is proportional to the number of significantly correlated genes in over-represented terms and the color code, from blue to red, indicates the significance level (p-value -log10 scale).

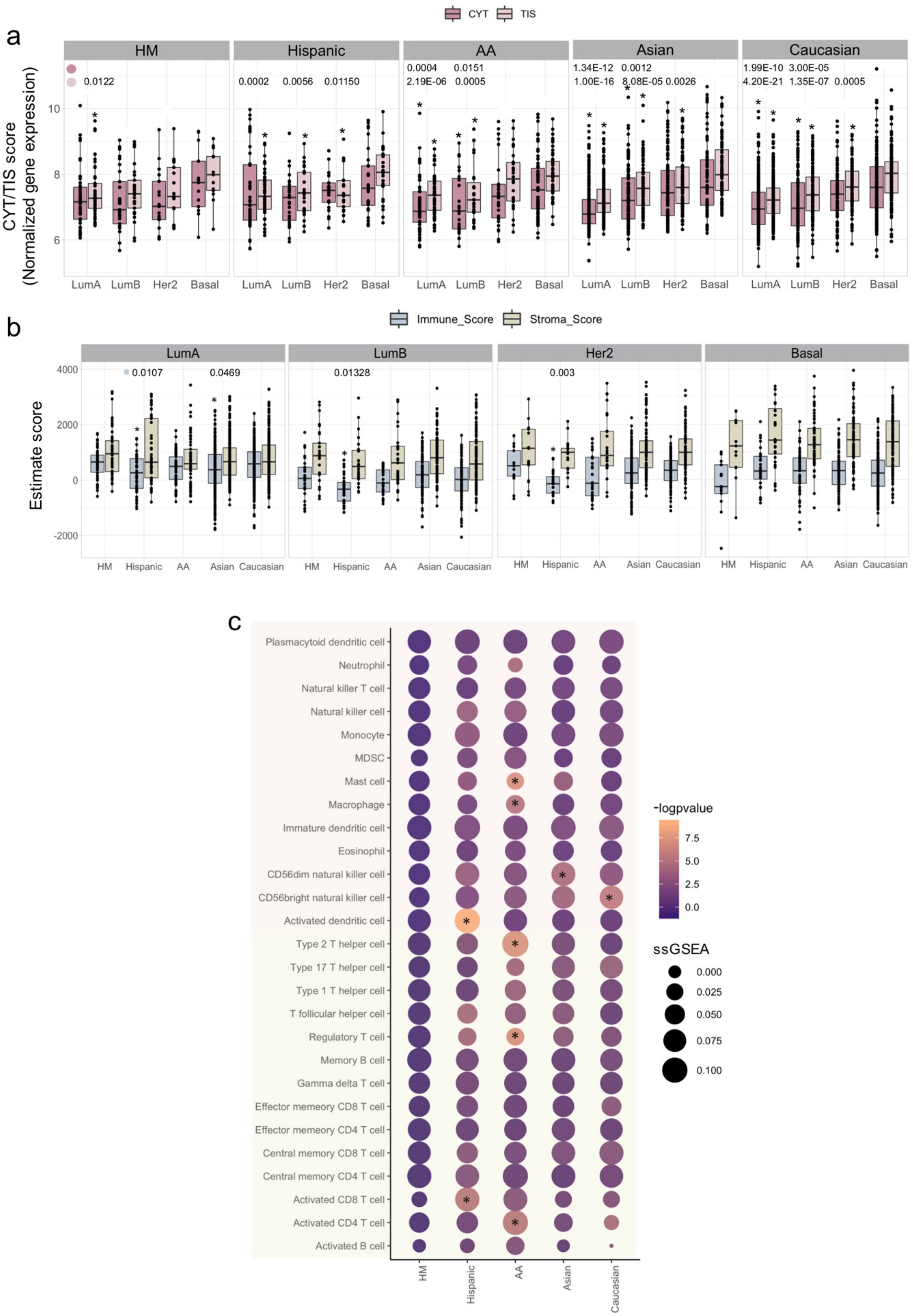

a
CYT
TIS
HM
Hispanic
AA
Asian
Caucasian
0.0122
0.0002 0.0056 0.01150
0.0004 0.0151
2.19E-06 0.0005
1.34E-12 0.0012
1.00E-16 8.08E-05 0.0026
1.99E-10 3.00E-05
4.20E-21 1.35E-07 0.0005
CYT/TIS score (Normalized gene expression)
LumA LumB Her2 Basal
b
Immune_Score
Stroma_Score
LumA
LumB
Her2
Basal
0.0107 0.0469
0.01328
0.003
Estimate score
HM Hispanic AA Asian Caucasian
c
Plasmacytoid dendritic cell
Neutrophil
Natural killer T cell
Natural killer cell
Monocyte
MDSC
Mast cell
Macrophage
Immature dendritic cell
Eosinophil
CD56dim natural killer cell
CD56bright natural killer cell
Activated dendritic cell
Type 2 T helper cell
Type 17 T helper cell
Type 1 T helper cell
T follicular helper cell
Regulatory T cell
Memory B cell
Gamma delta T cell
Effector memeory CD8 T cell
Effector memeory CD4 T cell
Central memory CD8 T cell
Central memory CD4 T cell
Activated CD8 T cell
Activated CD4 T cell
Activated B cell
HM
Hispanic
AA
Asian
Caucasian
-logpvalue
7.5
5.0
2.5
0.0
ssGSEA
0.000
0.025
0.050
0.075
0.100

Supplementary Fig. 11: Immune-landscape among ancestry-groups. a) Boxplot describing the distribution of CYT and TIS score among PAM50 subtypes in each ancestry group. significant differences against HM group (* p-value <0.05, HM as reference) estimated with two-tailed Wilcoxon rank-sum test. b) Boxplot describing the distribution of immune and stroma scores computed with Estimated algorithm among PAM50 subtypes in each ancestry group. Asterisks indicate significant differences against HM group (* p-value <0.05, HM as reference) in accordance to color legend, estimated with two-tailed Wilcoxon rank-sum test. c) Bubble plot sowing the normalized ssGESEA scores of adaptive (yellow square) and innate (purple square) infiltrated immune-cell populations among ancestry-groups. Color of the bubble represents -log10 p-value of the two-tailed Wilcoxon test between HM and each of the other ancestry-groups. Size of the bubble represents normalized ssGSEA score. Box plots in panel a-b represent median ± IQR (25th and 75th percentile) and whiskers correspond to maximum and minimum values. Statistics from panel c described at supplementary data 10. AA: African American, HM: Hispanic Mexican, LumA: Luminal A, LumB: Luminal B, CYT: cytolytic activity score, TIS: Tumor inflammation signature.
