# Supplementary material for: Comprehensive omic characterization of breast cancer in Mexican-Hispanic women
Source: Nat Commun. 2021 Apr 14;12:2245. doi: 10.1038/s41467-021-22478-5 (PMC8046804; doi:10.1038/s41467-021-22478-5)
Supplement: Supplementary file 3 — Description of Additional Supplementary Files [file 41467_2021_22478_MOESM3_ESM.pdf]

Description of Additional Supplementary Files
File Name: Supplementary Data 1
Description: Summary of clinical features and "omic" characterization of HM data-set and the descriptive statistics for all study variables (N=204)

File Name: Supplementary Data 2
Description: Information and clinical features of multi-ethnic publicly available data analyzed and the descriptive statistics for HM, TCGA, Asian Kan, Lace and SEER. (N= 4045, n= 339 AA, 855 Asian, 2427 Caucasian, 129 Hispanic non-Mexican, 257 Hispanic Mexico, 186 Nigerian), and two-sided Fisher exact test statistics of young and older patient across the analyzed samples. Statistics of fisher exact text for each comparison from Fig. 2 and

Supplematary Figure 3
File Name: Supplementary Data 3
Description: Tumor mutational burden (TMB) of evaluated tumors (N=1429 , 134 HM, 119 AA, 57 Asian TCGA, 250 Asian Japanese, 185 Asian Kan, 684 Caucasian)

File Name: Supplementary Data 4
Description: Cosmic mutational signatures (top 5) in tumors from diverse multi- ethnic data sets deconstructed with DeconstructSig and SigFit tools (N=568 , 52 AA, 30 Asian TCGA, 94 Asian Kan , 323 Caucasian, 69 HM): summary of signatures contribution, deconstructSig computed probability and incidence, SigFit computed probability and incidence. Only samples with a TMB over the median of each tumor collection were included in the analysis. Statistics of the assocaition between top 5 mutational signatures and ancestries, and immunochemestry and TMB features (Data from Sup Fig. 6b-c) .

File Name: Supplementary Data 5
Description: Mutation information (maf file) and lists of significant mutations computed with MutSig of each analyzed ethnic-groups. P-values were computed by testing if the observed mutations in a gene significantly outpace the expected background model. p values were adjusted by False Discovery Rates.

File Name: Supplementary Data 6
Description: Identified cancer driver mutations on HM profiles tumors computed with oncodriveMUT, their frequency along human ethnicities, the total number and two-sided Fisher exact test statistics analysis of driver mutations in each analyzed data-set.

File Name: Supplementary Data 7
Description: GISTIC summary of all lesions in each wide peak (HM, AA TCGA, Caucasian TCGA and Asian TCGA). GISTIC p-values for each marker were determinate by comparing the score at each locus to a background score distribution computed by random permutation of the marker location in each sample. P-values were then adjusted for multiple-hypothesis testing using the Benjamini-Hochberg method.

File Name: Supplementary Data 8
Description: GISTIC confidence genes (q value> 0.25) among ethnic-groups: amplifications and deletions.

File Name: Supplementary Data 9
Description: Supplementary Data 9: Coordinated altered mechanisms in evaluated tumors: TMB-TAF landscape, correlation matrix of cis-effects of SCNA events on mRNA expression patterns (Pearson correlation R.0.30, $p<0.05$) (logFC1.5, $p<0.05$) and MEMo results for HM profiled tumors (N= 886 , 117 AA, 57 Asian, 663 Caucasian, 49 HM)

File Name: Supplementary Data 10
Description: Supplementary Data 10: Normalized ssGSEA scores calculated to each immunecell population (Gene expression profile defined with Affymetrix array N=1672) and their statistical differences (two-tailed Wilcoxon test) among ethnic-groups and PAM50 intrinsic subtypes (HM cohort used as reference). Statistics from Figure 8c-d and Supplementary Figure 11c.
