# Supplementary material for: Comprehensive omic characterization of breast cancer in Mexican-Hispanic women
Source: Nat Commun. 2021 Apr 14;12:2245. doi: 10.1038/s41467-021-22478-5 (PMC8046804; doi:10.1038/s41467-021-22478-5)
Supplement: Supplementary file 14 — Reporting Summary [file 41467_2021_22478_MOESM14_ESM.pdf]

nature research

Corresponding author(s): Sandra L. Romero-Córdoba , sromero@iibiomedicas.unam.mx
Alfredo Hidalgo-Miranda, ahidalgo@inmegen.gob.mx

Last updated by author(s): Jan 28, 2021

Reporting Summary

Nature Research wishes to improve the reproducibility of the work that we publish. This form provides structure for consistency and transparency in reporting. For further information on Nature Research policies, see Authors & Referees and the Editorial Policy Checklist.

Please do not complete any field with "not applicable" or n/a. Refer to the help text for what text to use if an item is not relevant to your study.
For final submission: please carefully check your responses for accuracy; you will not be able to make changes later.

Statistics

For all statistical analyses, confirm that the following items are present in the figure legend, table legend, main text, or Methods section.

n/a	Confirmed	
☐	☒	The exact sample size (*n*) for each experimental group/condition, given as a discrete number and unit of measurement
☐	☒	A statement on whether measurements were taken from distinct samples or whether the same sample was measured repeatedly
☐	☒	The statistical test(s) used AND whether they are one- or two-sided<br>*Only common tests should be described solely by name; describe more complex techniques in the Methods section.*
☐	☒	A description of all covariates tested
☐	☒	A description of any assumptions or corrections, such as tests of normality and adjustment for multiple comparisons
☐	☒	A full description of the statistical parameters including central tendency (e.g. means) or other basic estimates (e.g. regression coefficient) AND variation (e.g. standard deviation) or associated estimates of uncertainty (e.g. confidence intervals)
☐	☒	For null hypothesis testing, the test statistic (e.g. *F*, *t*, *r*) with confidence intervals, effect sizes, degrees of freedom and *P* value noted<br>*Give P values as exact values whenever suitable.*
☒	☐	For Bayesian analysis, information on the choice of priors and Markov chain Monte Carlo settings
☒	☐	For hierarchical and complex designs, identification of the appropriate level for tests and full reporting of outcomes
☒	☐	Estimates of effect sizes (e.g. Cohen's *d*, Pearson's *r*), indicating how they were calculated

Our web collection on statistics for biologists contains articles on many of the points above.

Software and code

Policy information about availability of computer code

Data collection	No software was employed for data collection
Data analysis	1. Software used for mRNA expression profiles analysis:<br>R packages in R v.4.0.1 (http://www.r-project.org/)<br>Oligo R package v 1.46<br>ComBat function from SVA R package v3.36.0,<br>llimma R package v3.38.3,<br>biomaRt R package v2.38.0<br><br>2. Software used for Quality control of samples using expression<br>ESTIMATE R package v0.22.0,<br><br>3. Software used for Pam50 subtyping<br>pbcmc R package v1.6.0,<br>TNBCtype v.1.0 (http://cbc.mc.vanderbilt.edu/tnbc/)<br><br>4. Software used forAncestry proportion analysis<br>ADMIXTURE Software v1.3.0<br><br>5. Software used for Somatic mutation analysis<br>MutSigCV GenePattern module v1.3.01<br>TransVar v2.4.0 (https://bioinformatics.mdanderson.org/transvar/)<br>OncodriveMUT (https://www.cancergenomeinterpreter.org/home)<br>Maftools R package v1.8.10

Segmented R package v1.3-1
deconstructSigs R package v1.8.0,
SigFit R package v2.0,

6. Software used for Copy Number analysis
Genotyping Console 4.0, Affymetrix Inc.
Programs used as part of the Affymetrix SNP6 Copy Number Inference Pipeline implemented in Genepattern GenePattern 3.9.9(http://genepattern.broadinstitute.org/gp/pages/index.jsf):
SNPFileCreator v1.0,
Birdseed v.1.0,
TangentNormalization and
ParallelCBS v1.0
GISTIC v2.0.23

7. Other softwares employed for annotations and functional characterization
MEMo v1.1
GSVA R package v1.36.2
enrichR R package v2.1,

For manuscripts utilizing custom algorithms or software that are central to the research but not yet described in published literature, software must be made available to editors/reviewers. We strongly encourage code deposition in a community repository (e.g. GitHub). See the Nature Research guidelines for submitting code & software for further information.

Data

Policy information about availability of data

All manuscripts must include a data availability statement. This statement should provide the following information, where applicable:

- Accession codes, unique identifiers, or web links for publicly available datasets
- A list of figures that have associated raw data
- A description of any restrictions on data availability

1. Datasets supporting the findings of this study are available from:
Gene Expression data generated in this study have been deposited in the GEO database under accession code GSE86374 [https://www.ncbi.nlm.nih.gov/geo/query/acc.cgi?acc=GSE86374]
SNP array data generated in this study have been deposited in the GEO database under accession code GSE87048 [https://www.ncbi.nlm.nih.gov/geo/query/acc.cgi?acc=GSE87048], respectively.

2. Gene Expression Datasets used in this study are available in the GEO database under the following accession codes:
GSE78958 [https://www.ncbi.nlm.nih.gov/geo/query/acc.cgi?acc=GSE78958],
GSE16716 [https://www.ncbi.nlm.nih.gov/geo/query/acc.cgi?acc=GSE20194],
GSE20271 [https://www.ncbi.nlm.nih.gov/geo/query/acc.cgi?acc=gse20271],
GSE37751 [https://www.ncbi.nlm.nih.gov/geo/query/acc.cgi?acc=GSE37751],
GSE48390 [https://www.ncbi.nlm.nih.gov/geo/query/acc.cgi?acc=GSE48390],
GSE54002 [https://www.ncbi.nlm.nih.gov/geo/query/acc.cgi?acc=GSE54002],
GSE15852 [https://www.ncbi.nlm.nih.gov/geo/query/acc.cgi?acc=GSE15852],
GSE2109 [https://www.ncbi.nlm.nih.gov/geo/query/acc.cgi?acc=gse2109],
GSE75678 [https://www.ncbi.nlm.nih.gov/geo/query/acc.cgi?acc=GSE75678],
GSE113184 [https://www.ncbi.nlm.nih.gov/geo/query/acc.cgi?acc=GSE113184],
GSE59595 [https://www.ncbi.nlm.nih.gov/geo/query/acc.cgi?acc=GSE59595]

3. Public Repositories and clinical information Tables from third parties used in this study
Somatic mutations and annotation data from HM samples can be retrieved from cbioportal under the URL [https://cbioportal-datahub.s3.amazonaws.com/brca_broad.tar.gz] and from the dbGAP database [https:// https://www.ncbi.nlm.nih.gov/gap/] under accession number phs001250. v1.p1 [https://www.ncbi.nlm.nih.gov/projects/gap/cgi-bin/study.cgi?study_id=phs001250.v1.p1] (upon authorization request)

Somatic mutations and annotation data from TGCA breast cancer samples are available in Firebrowse database under the URL [http://gdac.broadinstitute.org/runs/stddata__2016_01_28/data/BRCA/20160128/gdac.broadinstitute.org_BRCA.Mutation_Packager_Oncotated_Calls.Level_3.2016012800.0.0.tar.gz]

Copy number data from TGCA breast cancer samples are available in Xenabrowser portal under the URL [https://xenabrowser.net/datapages/?dataset=TCGA-BRCA.cnv.tsv&host=https%3A%2F%2Fgdc.xenahubs.net&removeHub=https%3A%2F%2Fxena.treehouse.gi.ucsc.edu%3A443]

Clinical and molecular subtype data from breast cancer samples can be retrieved from cbioportal database under the following URLs:
METABRIC [https://www.cbioportal.org/study/clinicalData?id=brca_metabric]
Metastatic Breast Cancer (INSERM, PLoS Med 2016) [https://www.cbioportal.org/study/clinicalData?id=brca_igr_2015]

Clinical and molecular subtype from Nigerian breast cancer samples (Pitt et al, 2018) are available as supplementary information in the URL [https://static-content.springer.com/esm/art%3A10.1038%2Fs41467-018-06616-0/MediaObjects/41467_2018_6616_MOESM4_ESM.xlsx]

Clinical and molecular subtype from Asian breast cancers samples (SMC Kan et al, 2018) are available as supplementary information in the URL [https://www.nature.com/articles/s41467-018-04129-4#Sec24]

Clinical and molecular subtype from Japanese breast cancer samples (Hatakeyama et al, 2019) are available as supplementary information in the URL (https://onlinelibrary.wiley.com/action/downloadSupplement?doi=10.1111%2Fcas.14087&file=cas14087-sup-0002-TableS1.xlsx)

4. URL links to databases used in this study
cancer hotspot database [http://cancerhotspots.org]
OncoKB [https://www.oncokb.org/]
Cancer Genome Interpreter [https://www.cancergenomeinterpreter.org/home]
COSMIC project v2[www.cancer.sanger.ac.uk/cosmic/signaturesnd]

Field-specific reporting

Please select the one below that is the best fit for your research. If you are not sure, read the appropriate sections before making your selection.

- [x] Life sciences
- [ ] Behavioural & social sciences
- [ ] Ecological, evolutionary & environmental sciences

Life sciences study design

All studies must disclose on these points even when the disclosure is negative.

Sample size

The present study includes tumor samples from 204 Mexican patients diagnosed with primary breast cancer, without a second tumor and treated with adjuvant therapy at the Institute of Breast Diseases (FUCAM) from 2008-2012. No sample size calculation was done. However this sample size is concordant with those included in different works in which association between mutational and molecular alterations and ancestry in different populations has been encountered with sufficient power. Even more, we have leveraged various public datasets to validate and increase the statistical significance of the associations and differences found in our study

Data exclusions

Our original collection samples comprised about 400 Samples, but those with poor DNA quality control or tumor content values less than 60% were excluded right from the beginning . The reason behind this decision was to avoid the possible bias these samples could have introduced to our analysis and conclusions, due precisely to the fact that DNA or RNA quality does not comply with the minimum requirements to be adequately processed and to the impossibility to reliably ensure that such material originates from cancer cells within such samples. For PAM50 intrinsic subtype frequency and related analysis, samples classified as normal like where excluded to prevent the effect due to possible normal cell majority content

Replication

The large quantity of samples and the high costs of performing experiments in the "omics" contexts, precludes the possibility of obtaining technical replicates from each patient. However, as a mean to ensure the reproducibility, we leveraged various public datasets with which were included to validate, compare and increase the power of our own results and all data produced has been made publicly available in the indicated repository or as supplementary data within our manuscript. Furthermore, Our experimental pipeline was conducted with the use of standard and robust softwares which, in general, includes a validation method consisting of permutation processes or the application of statistical tests to asses significance. We sought to select stringent cutoffs for the selection of variables and samples of interest. Additionally, where applicable, our results were cross-validate by confronting the outputs produce by different algorithms.

Randomization

No randomization was performed, given that our study is observational and the fact the our analysis were conducted on various sample collections from different sources.

Blinding

Researchers blinding was not applicable, given the observational nature of our research and the fact the our analysis were conducted on various sample collections from different sources.

Reporting for specific materials, systems and methods

We require information from authors about some types of materials, experimental systems and methods used in many studies. Here, indicate whether each material, system or method listed is relevant to your study. If you are not sure if a list item applies to your research, read the appropriate section before selecting a response.

Materials & experimental systems

n/a	Involved in the study	
☐	☒	Antibodies
☒	☐	Eukaryotic cell lines
☒	☐	Palaeontology
☒	☐	Animals and other organisms
☐	☒	Human research participants
☒	☐	Clinical data

Methods

n/a	Involved in the study	
☒	☐	ChIP-seq
☒	☐	Flow cytometry
☒	☐	MRI-based neuroimaging

Antibodies

Antibodies used

ER/RP pharmDX (Dako, Denmark, K1904, ready-to-Use),
Estrogen receptor alpha (Dako, Denmark, M7047, 1:35),
Progesterone receptor (Dako, Denmark, M3569, 1:50),
Ki67 (Dako, Denmark, M7240, 1:50, clone MIB-1)

	HercepTest (Dako, Denmark, K5204),<br>DAKO EGFR pharmDxTM kit (Dako, Denmark K1492, ready-to-Use)<br>anti-CK5/6 antibody (Dako, Denmark, M7237, 1:20, clone D5/16 B4),<br>cytokeratin 14 (Novocastra, NCL-LL002, 1:20, clone LL02),<br>cytokeratin 17 (Dako, Denmark M7046, 1:2o, clone),<br>Claudin 1 (Abcam, UK, AB15099, 1:50),<br>Claudin 3 (Abcam, UK, AB15102, 1:50)
Validation	ER/RP pharmDX: https://www.agilent.com/en/product/pharmdx/herceptest-kits https://www.agilent.com/cs/library/usermanuals/public/28630_herceptest_interpretation_manual-breast_ihc_row.pdf<br>Estrogen receptor alpha: https://www.agilent.com/en/product/immunohistochemistry/antibodies-controls/primary-antibodies/progesterone-receptor-(concentrate)-76579<br>Progesterone receptor: https://www.agilent.com/en/product/immunohistochemistry/antibodies-controls/primary-antibodies/progesterone-receptor-(concentrate)-76579<br>: https://www.agilent.com/en/product/immunohistochemistry/antibodies-controls/primary-antibodies/ki-67-antigen-(concentrate)-76646<br>HercepTest: https://www.agilent.com/en/product/pharmdx/herceptest-kits https://www.agilent.com/cs/library/usermanuals/public/28630_herceptest_interpretation_manual-breast_ihc_row.pdf<br>DAKO EGFR pharmDxTM kit: https://www.agilent.com/en/product/pharmdx/egfr-pharmdx-kits/egfr-pharmdx-kit-for-manual-use-76902<br>anti-CK5/6 antibody: https://www.agilent.com/en/product/immunohistochemistry/antibodies-controls/primary-antibodies/cytokeratin-5-6-(concentrate)-76644<br>cytokeratin 14 (Novocastra, NCL-LL002, 1:20, clone LL02): https://www.labome.com/product/Leica-Biosystems/NCL-L-LL002.html<br>cytokeratin 17: https://www.agilent.com/en/product/immunohistochemistry/antibodies-controls/primary-antibodies/cytokeratin-17-(concentrate)-76623<br>Claudin 1: https://www.abcam.com/claudin-1-antibody-ab15098.html<br>https://www.abcam.com/claudin-3-antibody-ab15102.html<br>Claudin 3: https://www.abcam.com/claudin-3-antibody-ab15102.html

Human research participants

Policy information about studies involving human research participants

Population characteristics	This information is described as a table (ST1.xlsx) within the manuscript
Recruitment	For the HM cohort, patients diagnosed with primary breast cancer, without a second tumor and treated with adjuvant therapy were recruited by practitioner oncologists at the Institute of Breast Diseases (FUCAM) from 2008-2012. All participants gave their consent to enter our research protocol after being properly informed. For the rest of the sample collections from other studies included within this one, we refer to the original source publication. The fact that our samples were collected in a mono-center scheme represents a possible source of bias given that FUCAM provided services to vulnerable population covered by Seguro Popular de Salud (Popular Health Insurance), which is aimed to extend health care coverage to the Mexican population, and gratuity interventions, therefore, patients within our collection might over-represent particular socio-economical, cultural and environmental conditions. Furthermore, difficulties at gathering information regarding such conditions for all patients, impeded us to correct for all this variables which, as pointed out in our conclusions is a paramount task to address in further studies.
Ethics oversight	Ethics and Research committees of the National Institute of Genomic Medicine and FUCAM Institute in Mexico City (CE2009/11) and regulatory review bodies

Note that full information on the approval of the study protocol must also be provided in the manuscript.

This checklist template is licensed under a Creative Commons Attribution 4.0 International License, which permits use, sharing, adaptation, distribution and reproduction in any medium or format, as long as you give appropriate credit to the original author(s) and the source, provide a link to the Creative Commons license, and indicate if changes were made. The images or other third party material in this article are included in the article's Creative Commons license, unless indicated otherwise in a credit line to the material. If material is not included in the article's Creative Commons license and your intended use is not permitted by statutory regulation or exceeds the permitted use, you will need to obtain permission directly from the copyright holder. To view a copy of this license, visit http://creativecommons.org/licenses/by/4.0/
